# Correction to “Artificial Intelligence in Natural Product Drug Discovery: Current Applications and Future Perspectives”

**DOI:** 10.1021/acs.jmedchem.5c01675

**Published:** 2025-06-27

**Authors:** Amit Gangwal, Antonio Lavecchia

In addition to the references previously cited in the text, we have incorporated an additional reference (ref 1 in this correction) to properly acknowledge a prior comprehensive review on the same topic. This reference should be inserted at the following locations:

- **Page 3952, Column 1**, Section 3.1 “AI in NP Target Prediction and Deorphanizing”: The new reference should be inserted after the sentence: “As these substances are of natural origin, they are more likely to interact effectively with transporter systems, facilitating their delivery to target sites.”
- **Page 3953, Column 2**, Section 3.2 “AI in NP Genome and Metabolome Mining”: The new reference should be inserted after the sentence: “AI has been increasingly utilized to predict biosynthetic genes and metabolite structures from sequence or spectrum data, significantly accelerating the discovery of NPs.”
- **Page 3959, Column 1**, Section 3.5 “AI in Structural Characterization (Chemical Structure Prediction) of NPs”: The new reference should be inserted after the sentence: “Recent innovations include microcrystal electron diffraction (MicroED), which accelerates the investigation of sub-micrometer-sized crystals of chemical compounds and potentially expedites structure elucidation.”
